# Risk factors for childhood illness and death in rural Uttar Pradesh, India: perspectives from the community, community health workers and facility staff

**DOI:** 10.1186/s12889-021-12047-2

**Published:** 2021-11-06

**Authors:** Kanchan Srivastava, Ranjana Yadav, Lorine Pelly, Elisabeth Hamilton, Gaurav Kapoor, Aman Mohan Mishra, Parwez Anis, Maryanne Crockett

**Affiliations:** 1 India Health Action Trust, 404 - 4th Floor, 20-A Ratan Square, Vidhan Sabha Marg, Lucknow, Uttar Pradesh 226001 India; 2 University of Manitoba, Institute for Global Public Health, R070 Med Rehab Building, 771 McDermot Avenue, Winnipeg, Manitoba R3E 0T6 Canada; 3 Departments of Pediatrics and Child Health, Medical Microbiology and Infectious Diseases and Community Health Sciences, University of Manitoba, Winnipeg, Canada

**Keywords:** Child morbidity and mortality, Risk factors, Perceived risk

## Abstract

**Background:**

Uttar Pradesh (UP), India continues to have a high burden of mortality among young children despite recent improvement. Therefore, it is vital to understand the risk factors associated with under-five (U5) deaths and episodes of severe illness in order to deliver programs targeted at decreasing mortality among U5 children in UP. However, in rural UP, almost every child has one or more commonly described risk factors, such as low socioeconomic status or undernutrition. Determining how risk factors for childhood illness and death are understood by community members, community health workers and facility staff in rural UP is important so that programs can identify the most vulnerable children.

**Methods:**

This qualitative study was completed in three districts of UP that were part of a larger child health program. Twelve semi-structured interviews and 21 focus group discussions with 182 participants were conducted with community members (mothers and heads of households with U5 children), community health workers (CHWs; Accredited Social Health Activists and Auxiliary Nurse Midwives) and facility staff (medical officers and staff nurses). All interactions were recorded, transcribed and translated into English, coded and clustered by theme for analysis. The data presented are thematic areas that emerged around perceived risk factors for childhood illness and death.

**Results:**

There were key differences among the three groups regarding the explanatory perspectives for identified risk factors. Some perspectives were completely divergent, such as why the location of the housing was a risk factor, whereas others were convergent, including the impact of seasonality and certain occupational factors. The classic explanatory risk factors for childhood illness and death identified in household surveys were often perceived as key risk factors by facility staff but not community members. However, overlapping views were frequently expressed by two of the groups with the CHWs bridging the perspectives of the community members and facility staff.

**Conclusion:**

The bridging views of the CHWs can be leveraged to identify and focus their activities on the most vulnerable children in the communities they serve, link them to facilities when they become ill and drive innovations in program delivery throughout the community-facility continuum.

**Supplementary Information:**

The online version contains supplementary material available at 10.1186/s12889-021-12047-2.

## Background

Globally, rates of child mortality are decreasing with the total number of under-five (U5) deaths dropping from 14.2 million in 1990 to 6.2 million in 2018 [1]. Of the 6.2 million global U5 deaths in 2018, close to 900,000 occurred in India [2] and many districts in India are forecasted to miss the U5 mortality targets for 2030 [3]. Therefore, India must accelerate progress in child survival. Pneumonia and diarrhoea remain two of the leading causes of death in U5 children in India, accounting for 15.9 and 9.3% of deaths, respectively [4]. In Uttar Pradesh (UP), with one of the highest U5 mortality rates in India at 47 per 1000 live births [5], pneumonia and diarrhoea are responsible for more than 60,000 preventable deaths annually [6, 7].

There is significant overlap between risk factors for childhood pneumonia and diarrhoea which led to advocacy for integrated programs. Shared risk factors include short duration of exclusive breastfeeding, vitamin A deficiency, zinc deficiency, under-immunization, malnutrition, prematurity, low birth weight, recent measles infection, co-morbidity and low socio-economic status of the caregiver [8, 9]. It has also been observed that pneumonia and diarrhoea deaths are associated with multiple risk factors, such as poverty, undernutrition, poor hygiene and lower-resourced home environments [9].

For severe pneumonia, low birth weight, lack of exclusive breastfeeding, crowding, indoor air pollution, suboptimal immunization, undernutrition and HIV infection were the most predictive risk factors related to the development of illness [10]. Another recent study looked at risk factors for pneumonia in the Western Pacific region and highlighted lack of breastfeeding, cigarette smoke, air pollution exposure, malnutrition and conditions of poverty among the major determinants [11]. Poor vaccination coverage, sub-optimal pneumonia case management and delay in seeking treatment have also been highlighted as risk factors for pneumonia in children [12, 13].

For severe diarrhoea, specifically, high number of stools, not being breastfed, young age and developing diarrhea in the rainy season are risk factors for prolonged diarrhea [14]. A similar study noted that shared drinking water sources and early introduction of supplemental foods were significant risk factors for childhood diarrhoea [15].

Key individuals and groups must identify vulnerabilities and risk factors for illness and death in young children so that appropriate care can be provided at the right time. Perception by caregivers of childhood illnesses including severity and how the illnesses should be treated has been demonstrated to impact timely and appropriate care-seeking. In Pakistan, the decisions to access care for children who are sick with pneumonia or diarrhoea was driven by how severe the illness was perceived to be, personal or community experience with the illness, beliefs and experiences with home remedies and the influence of the elder generation in the community [16]. Similar findings have been described in other settings [17, 18]. However, there is sparse research on how the caregivers perceived underlying risk factors that are related to a higher likelihood of their children experiencing illness.

The role of community health workers (CHWs) in improving health outcomes at the grassroots level has been globally recognized [19]. In general, CHWs often belong to the same community of users or live near the same community where they provide their services. In a systematic review of CHW effectiveness, it was shown that CHWs can contribute to the health system by “reducing inequalities in health care for marginalized populations, providing education and some curative health services, and having an essential role of liaising between the community and facility-based services provided by more skilled workers” [20]. Some studies have looked at recognition and response to clinical danger signs from the perspective of caregivers and health workers [21, 22]. However, to prioritize the most vulnerable children, it is imperative to delineate more broadly how risk factors for childhood illness and death are perceived by different groups in the care pathways of sick young children.

## Methods

This study was completed as part of a larger Child Health Program to understand how risk factors for childhood illness and death are understood by community members, CHWs and facility staff. This study used two qualitative research methods: focus group discussions (FGDs) and semi-structured in-depth interviews (IDIs).

### Study context

The study was implemented by the Uttar Pradesh Technical Support Unit (UP-TSU) as part of a larger Child Health program which aimed to reduce the case fatality rates of pneumonia and diarrhoea among U5 children by improving the quality of community and block level facility management of childhood illness. The state of UP is comprised of 75 districts and 822 blocks at the sub-district level [23]. The initial footprint of the project was in 15 blocks within three districts in UP. The UP-TSU works closely with the Government of Uttar Pradesh to support the implementation of government programs in the areas of Reproductive, Maternal, Newborn, Child and Adolescent Health.

The CHW cadre in this study is composed of Accredited Social Health Activists (ASHAs) and Auxiliary Nurse Midwives (ANMs). ASHAs were instituted by the Government of India's Ministry of Health and Family Welfare as part of the National Rural Health Mission to mobilize, counsel and support community members to seek health services [24]. ANMs are village-level female health workers in India who are known as the key link between the community and the health facility. ANMs are regarded as the first-level in the health system organization pyramid [25].

### Study area and participants

This study was conducted in one block in each of the three districts initially covered by the Child Health Program with the three blocks purposively selected as they continued to have program personnel in place at the block level. Medical officers (MOs) and staff nurses (SNs) from the Community Health Centre (CHC) in each identified block were purposively selected and interviewed based on their availability on the day of data collection. There was only one CHC per block. Interviews occurred in a private room at the CHC. For the FGDs, two sub-centres were purposively selected so that, for each block, the study included one sub-centre that was located close to the CHC and one that was farthest from the CHC in order to maximize geographic variability within the block. An individual sub-centre was treated as a unit of data collection for the FGDs. For each selected sub-centre, all ASHAs in that sub-centre were invited to participate in the FGD. The block level program staff member visited the selected sub-center on the day prior to data collection and with the support of the ASHA identified the mothers of U5 children and male heads of households (HH) with U5 children from different geographies within the village in which the selected sub-centre was located. The selection of participants for FGDs was based on convenience, their desire to participate and the geographic location of their residence in the village. All of the participants were approached in person for consent to participate in the study. A social mapping exercise was also done with the respondents to ensure geographical representativeness. The FGDs were conducted in a quiet space in a public building near to the sub-centre and settings included the Panchyat building, the Anganwadi centre or a school. One FGD occurred in the home of one of the ASHAs. Study team members who were not interviewing or taking notes monitored the doors of the FGD space to help ensure confidentiality.

To explore the research objectives, the participants within each individual FGD were selected based on homogeneous characteristics [26]. Selecting homogeneous FGD participants is intended to allow deeper description of a particular subgroup, reduce variation, enable analysis and facilitate group interviewing [27]. The homogeneous characteristics included being an ASHA, an ANM, a mother with an U5 child and a male HH with a U5 child. Each of these four groups participated in separate FGDs. Diversity of perspective was maintained by having different groups participate in the FGDs and having geographic representation from within the village. In total, 21 FGDs and 11 in-depth interviews were conducted with the selected participants as shown in Table 1; the average size of each FGD was eight participants. ANMs were also included in in-depth interviews if they were working at the block facility level. None of the individuals approached to participate declined or withdrew from participation.

**Table 1:** Number of participants in focus group discussions and interviews, by study block and group

District	HH with U5 child	Mother of U5 child	ASHA	ANM (FGD)	ANM (IDI)	Medical officer	Staff Nurse
	Focus Group Discussions (FGD)				In-Depth Interviews (IDI)		
1	17	14	14	7	0	3	2
2	19	21	14	10	1	2	1
3	16	19	13	5	1	0	1
Total	**52**	**54**	**41**	**22**	**2**	**5**	**4**

*ASHA* Accredited Social Health Activist, *ANM* Auxiliary Nurse Midwife, *FGD* focus group discussion, *HH* head of household, *IDI* in-depth interview, *U5* under-five

### Data collection and analysis

The data collection and analysis team was a mixed group of researchers with academic backgrounds that included Anthropology, Sociology, Public Health and Medicine. KS and RY were lead researchers for data collection and analysis and conducted the FGDs and interviews. KS holds a PhD in Anthropology and RY holds a MSc in Anthropology. Both have been working and conducting public health research in UP for more than 10 years and were employed as research specialists for India Health Action Trust at the time of the study. LP and MC are both pediatricians and public health professionals. AMM and GK have credentials in public health and supported data collection and analysis.

A topic guide was developed and piloted prior to the FGDs and interviews. To elicit participants’ views and experiences, the topic guide prompted lines of inquiry regarding knowledge around illness among children, perceived vulnerability for childhood illness and challenges and strategies for identifying and prioritizing areas with increased concentrations of children who were perceived as being vulnerable. Examples of an interview guide and a focus-group discussion guide are shown in supplementary file 1.

Data was collected for the study in September 2017. The interview and FGD length ranged from 23 to 77 min. All of the interviews and FGDs were audio-recorded with consent given by all participants. Notes were also made during the FGDs. The audio-recordings were transcribed and translated into English. Dedoose (Version 8.0.35) [28] software was used for coding the translated material. The transcripts were not verified with the participants. Saturation was largely achieved following data collection in the second block but additional local terms for childhood illnesses were added in the third block. Content analysis was conducted to highlight emerging patterns and themes that were derived from the data [29, 30].

Multiple steps were taken during the data collection to improve the validity of the study results. A topic guide was developed and used to ensure that a similar range of topics were discussed with all participants. To enhance the reliability of the coding framework, the three coders from the research team were initially assigned the same transcripts to code until consistency was obtained. Once intra- and inter-coder consistency had been achieved and the code sheet was standardized, each of the coders were assigned different transcripts for coding. In addition, 14 transcripts were double coded by two senior researchers to confirm the quality of the coded transcripts. The study codes and sub-codes derived from the data are available in supplementary file 2. Data collection was completed in three study areas according to the study plan; however, data saturation was achieved early. The participants did not provide feedback on the findings.

## Results

The risk factors perceived to have a significant role in childhood illness in rural UP vary according to the perspective of those who make decisions for a sick child in their care-seeking journey: community members (mothers and male heads-of-households with U5 children), community health workers (CHWs; ASHAs and ANMs) and facility staff at public health facilities (medical officers and staff nurses). The spectrum of perspectives is discussed in categories of convergent views (similar perspectives among all groups), mixed views (CHWs hold similar views to one of the groups) and divergent views (different perspectives among all groups). A summary of the results is shown in Table 2.

**Table 2:** Convergent, mixed and divergent explanatory perspectives on key risk factors for childhood illnesses

Themes	Perceived Risk Factors	Explanations		
		***Community***	***Community Health Worker***	***Facility Staff***
**Convergent**	Seasonality	Specific seasonal health problems e.g. diarrhea in monsoon season and pneumonia in winter; fever, cold and cough attributed to seasonal changes.		
	Lack of attentiveness	Prolonged absence of mother from home; families with many children results in compromised care of infants thus contributing to infections.		
	Social - Occupational factors	There are some occupations which are specific to certain caste, religion or geographical locality e.g. piggery, poultry, butchery, working in brick kilns. These occupations make families including their children prone to various adverse health conditions.		
**Mixed**	Gender	Girls are stronger by birth and nature hence they fall sick less often.	Girls are stronger than boys but both fall sick equally. Negligence of girl children in the community affect their care.	Negligence of girl children in the community might affect their care.
	Hygiene	Toddlers require close supervision and are more exposed to dirt and mud.	Household level lack of hygienic practices affect the child’s health.	
	Health of the mother	Mother’s poor health might lead to certain problems in child care but does not necessarily directly cause illness.	Mother’s health during pregnancy and her routines until the baby is breastfeeding determine the child’s health.	Mother’s health problems during pregnancy, e.g. anemia, genetic conditions determine the child’s health.
	Physical and biological factors of child	Health conditions: Weak (*kamjor)* children tend to fall sick frequently. Frequent episodes of illness in a child increases the risk of vulnerability.	Health factors: Newborn, birth weight, gestational age at birth and immunization status make children sick.	CHW viewpoint plus hereditary and congenital diseases as risk factors.
	Environment	Pollution, increased practices of food adulterations, use of chemicals in crops, etc. Improved clean water supply but occasional contamination by sewage makes it unfit for drinking.	Lack of safe and clean drinking water in the community; tendency to use untreated water.	
**Divergent**	Nutritional	Giving thick milk to a baby affects digestion; a child who eats frequently defecates for the whole day.	Complimentary feeding is either initiated early in poor families or later in well-off families. Both these factors make the child at increased risk of malnutrition (*kuposhan).*	Community practices encouraged by media advertisements like initiation of powdered milk, giving diluted milk and using packaged food/ juices lead to an inadequately nourished child (*kuposhit)*.
	Financial status and literacy	Households with low education often have low resources which can lead to delayed or poor quality care seeking.	Households with low education are difficult to counsel and they are hesitant to accept improved health practices.	Households with financial constraints cannot afford timely and quality care seeking.
	Location of habitation	Lack of accessibility in terms of distance from health facilities as well as poor transportation conditions, e.g. bad roads, discourage people from prompt care seeking.	Villages that are generally densely populated and are prone to water logging and flood are often predisposed to unhygienic conditions thus leading to outbreaks of many health problems.	The hard to reach geographical areas and poor housing conditions aggravate the risk of falling sick.

Table 2 Convergent, mixed and divergent explanatory perspectives on key risk factors for childhood illnesses.

### Convergent perceptions of risk factors

The risk factors for childhood illnesses that were perceived in a similar fashion by community members, CHWs and staff at public health facilities were the following: seasonality, lack of attentiveness from caregivers and social-occupational factors.

#### Seasonality

Certain health problems in children, such as diarrhoea, cold and cough and pneumonia, were strongly perceived to be associated with seasonality. CHWs explained that when the seasons change there are frequent episodes of illness and every child is at equal risk of falling sick.

*“When the weather starts shifting suddenly, it is important to be careful, both for the adults and the young ones. The weather at this time is such that it keeps fluctuating between hot or cold. Even the adults keep falling sick with something or the other. So obviously, the children are more vulnerable.”* (CHW).

Facility staff and members of the community expressed similar opinions that throughout the year there are specific seasons or months when the prevalence of illnesses is higher.

*“Between October and March - when it gets too cold - and then in the summers during which diarrhoea is very common. These two diseases, diarrhoea at this time and then pneumonia, cough etc. that happens October onwards.”* (Community)

#### Lack of attentiveness from caregivers

Based on their day-to-day experiences during household visits or interactions with the families in the community, the CHWs expressed that generally mothers are the best caregivers for young children. Hence their presence or absence has an impact on the health of the child. In some households, the mother had an occupational engagement and was away from home for the whole day which sometimes required young children to be left in the care of their siblings. Some of the CHWs empathised with working mothers who were not able to look after their children during the day as the CHWs have duties that can take them outside the home for the entire day.

In other households, the mother may be at home but pay less attention to the child than the ideal.

*“It happens so often that the child is eating rice and there are flies all over it, the mother does not even care about this. I had seen a child during pulse polio work … there were about a hundred flies on his mouth. I even told the mother … I could not see his lips; I had to remove the flies to give him the drops.”* (CHW)

Facility health providers also perceived that the dependency of a child is high during the first five years so lack of attention would affect their health. Community members explained that families with many children are not able to provide care to all the children and the care of one child gets compromised in the care of another child. They expressed that having many children will make them overburdened with their responsibilities which affects their ability to maintain proper hygiene and nutrition.

*“In [specific communities], children are born at a higher frequency. There are six or seven children there. The mother also has a problem because of this. If she bathes one, the other is crying … so the problem is because of population*.*”* (Community)

#### Social-occupational factors

There are some occupations which are specific to certain castes, religions or geographies such as *bhujwa* (roasters of rice and other snacks)*, dhune* (processers and weavers of cotton fibres)*,* those engaged in raising pigs and poultry, butchers, *bahelia* (hunters of birds)*, mushar* (catchers of rats and snakes) and workers in brick kilns. These occupations are perceived to make them and their children prone to various adverse health conditions.

There is a strong belief among the CHWs that there are specific castes and villages which generally do not keep the same level of hygiene as the rest of the community and they identify such areas as the sources of diseases which gradually spread to the rest of the localities.

*“They do not keep any difference between their own food and the food of the animals. They will eat in the same place and feed the animals there itself. There will be goats tied up in the same place where [there are] people. Even the food is cooked right there. The children are also set down in the same cots. The goat sits on the same cot too.”* (CHW)

Staff at public health facilities also highlighted that there are some occupations practiced by specific castes and/or religions in the community, e.g. those engaged in leather work, butchery or dealing with corpses, who they think are exposed to a higher risk of infections. Community members thought there had been a lot of improvement in their communities in terms of hygiene and lifestyle of the people. However, they echoed the viewpoint of CHWs about certain localities which maintain poorer hygiene than other people in the community.

### Mixed perceptions of risk factors

Perceptions of risk factors for childhood illness such as gender, hygiene, health of the mother, physical and biological factors in the child and the environment were mixed. The views of facility staff and community members were divergent about these factors but the views of CHWs overlapped with the views of one or sometimes both of the other groups.

#### Gender

Community members expressed that, in comparison to boys, girls fall sick less often. This is explained to be because girls are stronger by birth and, by their nature, they are more diligent in taking care of themselves and will maintain better hygiene and care.

“*Girls do not fall sick that often, boys do.”* (Community).

By contrast, facility staff shared that gender discrimination exists in the villages, but they have never seen any difference in the boys and girls in terms of falling sick more frequently.

The CHWs expressed mixed opinions on this issue. Most said that girls are stronger than boys but both fall sick equally.

“*Both boys and girls fall sick equally. But girls are stronger than boys.”* (CHW).

They expressed that if there were a difference, gender-based negligence in the community might be responsible for this difference because the community gives higher regard to boys than girls.

#### Hygiene

Community members, CHWs and staff at public facilities all placed household hygiene as one of the most important risk factors but the explanations differed between the facility staff and the CHWs compared to the community members. The CHWs discussed various instances where the child was exposed to increased risk for diarrhea due to lack of cleanliness such as the same water being used for cleaning cooking utensils, washing hands and feet and, at times, to collect urine. Facility staff attributed many of the health problems seen in young infants, particularly diarrhoea, to behaviours that led to lack of cleanliness in the households. Community members did discuss that unhygienic conditions might cause a young child to become ill but attributed it more to the developmental stage of the child. Toddlers are crawling everywhere and are more exposed to dirt and mud. Their personal hygiene is compromised and they frequently soil their clothes.

*“If the child is playing somewhere and they do not wash his hands before he starts eating something, then the dirt of the hand will go into the stomach and cause sickness … I have a daughter who crawls and moves around really fast, if I am not watching for even a minute, she will go from the bricked section to the mud house section … it is difficult to keep a watch on her and whatever she finds, she puts it in her mouth! My child eats a lot of mud. If he sits somewhere, if he even finds a clay pot, he starts eating it.”* (Community)

#### Health of the mother

The community members put more importance on the mother’s behavior towards the care of the child rather than her health status in determining the health of the child. They thought that a mother’s health was not always a predictor of a child’s health and that behavioural choices made a bigger difference.

*“Some mothers are weak but [their] children are very healthy. But some mothers are very healthy but [their] children are weak even though they get a good diet.”* (Community)

Facility staff strongly believed that the mother’s health has an impact on the child’s health with the health problems of the mother during pregnancy determining the child’s health.

*“If a woman is anaemic, she is definitely not going to have a healthy baby.”* (Facility Staff).

The CHWs held views that overlapped with those of the other two groups. While they expressed beliefs that the health of the mother was linked to a healthier baby, they also thought that specific behaviours of the mother had an impact.

“*If the mother has had some cough and cold, if she bathed at a wrong time or ate something wrong, the baby will get sick because of that. If that happens, he will fall sick again and again. But if she takes care of herself, her diet, her lifestyle, and then the baby will not fall sick*.” (CHW).

#### Physical and biological factors of the child

Community members generally identified risk factors as conditions of poor health that could be easily visually identified and raised the susceptibility for recurrent illness. *Kamjori* (weakness in the child) was a highly-perceived risk condition and referred to children who were visibly thin and notably less active than other children. Community members also identified children who were born premature and/or with low birth weight and thought that managing these children was just a matter of providing extra and proper care.

Facility staff recognized bio-medical risk factors such being in the newborn period, gestational age, birth weight, birth anomalies, genetic disorders and *kuposhan* (malnutrition) as the causes for childhood illness. CHWs expressed bio-medical views similar to the perspectives of the facility staff. They also emphasized the inherent vulnerability of the newborn period in addition to other factors such as birth weight, gestational age, health at the time of birth and vaccination status of both the mother and child.

“*New born babies … . Irrespective of whether they are born through caesarean or normal delivery, they need more care.”* (CHW)

#### Environment

CHWs and facility staff discussed that lack of clean water supply combined with the community’s use of untreated water led to infection and consequently affected the health of the community.

*“The biggest problem here is water. Ground water is available at seventy or eighty feet and people start drinking that water. That water, if kept for an hour turns yellow. It is from the hand pump.”* (CHW)

In a discussion of external environmental factors, the community members had strong perceptions that outside air pollution, unsafe drinking water and increased use of pesticides and chemicals in crops had deteriorated the quality of life of people in the community. The contamination of the water supply was seen to be more of an intermittent issue than continuous.

### Divergent perceptions of risk factors

This group of risk factors includes nutritional factors, financial status and literacy and location of habitation. The perceptions of each risk factor were different among all the groups.

#### Nutritional factors

CHWs were generally well-informed about malnutrition including causes and the effects on child health based on knowledge gained through routine trainings on health and nutrition. They thought that common community practices were responsible for inadequate nutritional status in children. The CHWs observed that in some households it is common practice to dilute milk prior to feeding it to children. They also observed that poor families start giving solid food earlier than recommended but also that relatively well-off families would initiate complementary feeding later than recommended.

“*The families of poor houses start giving food in four or five months. Those who are from affluent families start giving proper food to children after a year or so because they can afford external milk.*” (CHW)

Community members had different perceptions and some thought that characteristics of the child could lead to undernutrition observing that some children drink milk easily whereas others may not eat or drink much despite all efforts. They also expressed particular food-related beliefs that may affect the nutritional status of the child. This includes beliefs around the appropriate timing for hot and cold food, the importance of avoiding breastfeeding soon after work as this may cause the child to fall ill. There were also perceptions that if a child was fed undiluted milk or fed too frequently then this would lead to frequent passing of stools.

*“They should pay more attention, right? They should feed only thin milk. It should be as thin as water. At least up to three months of age, this is what should be fed to the infants. Otherwise, I believe that there is a ninety percent chance that the baby will fall sick.”* (Community)

The staff at public facilities recognized the problem of undernutrition but attributed the cause to the influence of the media on the choices made by families and their behaviours regarding complementary feeding.

#### Financial status and literacy

CHWs focused more on literacy and perceived that the households with lower education seem to be less accepting of counselling and advocacy messages. The households tended to be more resistant to accepting suggestions for change and to taking up health interventions.

*“First of all, education needs improvement … Those who are uneducated do not agree even if you try to counsel them. They say that children are meant to play in the mud … they say that when the children grow up, they will take care of themselves … those who are educated understand.”* (CHW)

The facility staff focused more on financial status than on literacy and observed that households with financial constraints cannot afford timely and quality care seeking. These financial constraints not only obstruct them in seeking medical services but also in obtaining basic care for their children.

*“There are many things but out of these, maintaining economic status is important. Without that, even though they may be aware, they will not be able to do anything. There are many people who are educated but they do not have the money so they cannot do anything while some uneducated people who have the money live a good quality life. It is all dependent on that.”* (Facility Staff)

Community members perceived both literacy and financial status as equally important risk factors for childhood illnesses. It was observed that households with low education often have low resources thus leading to delays in care-seeking and in the quality of care sought. Community members explained that availability of resources and the level of education helps in managing the problems that increase the risk for children, therefore, poor and illiterate families cannot provide better care to their children.

#### Location of habitation

The location where a family lives was perceived to be one of the risk factors for poor health in children but the explanations differed among the groups. Community members explained that a lack of accessibility due to distance from health facilities as well as poor transportation conditions, such as bad roads, discourage people from promptly seeking care and this affects the health of children. CHWs attributed the increased risk from location to places where villages are densely populated and prone to water logging and flooding which lead to unhygienic conditions and to many health problems.

“*The road in the eastern hamlet is very dirty, with water log [ging] and mud. All the diseases come from there. I went there for HBNC [home-based newborn care], and there’s no clean road to walk. When the animals defecate, it gets very dirty.”* (CHW)

The facility staff attributed risk to conditions of the housing itself in certain locations.

*“Wherever there are open spaces, the huts in the villages which is called Madha/Chappar [thatch] in the local term, such houses cannot stop the cold.”* (Facility Staff)

## Discussion

Perceptions regarding risk factors for severe illnesses in children vary among different stakeholders along the care-seeking pathway including community members, community health workers (CHWs) and clinical staff at public health facilities in UP. Interestingly, the interpretation of each identified risk factor as well as the importance placed on it was not uniform among the groups. Rather, the perceptions converge, diverge and overlap with each other in different areas. In the areas where the perceptions were mixed or diverged, the CHWs held views that often overlapped partially with either the community members or facility staff. The views of the community members and the facility staff were the most divergent. These findings are especially relevant within the context of a health system with ever-increasing expectations on CHWs to bridge the gap between communities and services provided at public health facilities.

The three primary areas which had converging perspectives on risk associations were seasonality, lack of attentiveness and socio-occupational factors. Community members, CHWs and facility staff offered similar explanations regarding why some children experienced severe illness. Although they agree in their explanations of causation, there were divergent approaches as to how these risks could be mitigated or managed. In the case of seasonality, all three groups associated certain seasons with some illnesses, e.g. rainy season/summer with diarrhea and winter with cold/cough and pneumonia. Since there is minimal guidance from CHWs and facility staff on preventative measures, households rely on their traditional wisdom such as using processed mustard oil with additives and herbs to treat colds and cough during the winter season. The community feels confident in their own traditional measures to treat colds and cough and may not recognize when illness has progressed and medical intervention is required. This represents a potential for delay in care-seeking and an opportunity for community education by the CHWs.

The three primary areas which had diverging perspectives on risk associations were nutrition, financial status/ literacy and location of habitation. For example, in terms of nutritional risk factors, the community members focused more on the concern of milk being too thick and compromising digestion and associating frequent feeding with frequent defecation. The CHWs noted that poor families started complimentary feeding too early and well-off families too late but that both behaviours led to children becoming malnourished. The health facility staff were more focused on the effect of media on the nutritional practices of the community. These divergent views highlight the difficulty in tackling behavior change regarding nutritional practices in young children with a single approach as others have noted [31].

The physical and biological characteristics of the child were an area with overlapping perspectives. The community members identified *kamjor* (weak) children as those who fell sick frequently and had higher risk for severe illness or death. The CHWs identified these children but attributed their risk to underlying issues such as being very young, being born early or of low birth weight and lack of immunization. The health facility staff perceived prematurity and low birth weight as risk factors in addition to hereditary and congenital diseases. These overlapping perspectives could be leveraged to improve care for children with a high level of vulnerability.

Reflecting on the intermediary position of the perspectives of CHWs in this study regarding risk factors for childhood illness and death, it is essential to understand how this important cadre can be leveraged for improving health in their communities. Depending on the program, CHWs function along a continuum between “service extender, cultural broker and social change agent” (p.3) [32]. CHWs in the Indian context have played an important role in improving community health outcomes. ASHAs are seen as link workers and their focus on rural and marginalized communities allows them to be seen as valued service providers [33]. The health system generally acknowledges CHWs’ role in program implementation but also undermines the socio-cultural and psychological belongingness of the CHWs which determines their approach to making decisions [34].

This study has a number of implications regarding the role of CHWs in improving child health in rural UP. The orientation and training programmes for CHWs generally focus on knowledge and skills for health interventions but have a smaller focus on engaging them within the community as members of that community. Ignoring the socio-cultural perspectives during one way, instruction driven training programmes might lead to reduced impact on behaviour change outcomes in the community as well as diminish the quality of counselling done by the health workers [35, 36].

The ASHA program in India has also been constrained by other factors. ASHAs’ limited knowledge about their role as activists, minimal and unreliable financial incentives and, most importantly, the poor quality of care at primary health centres has had negative consequences on the credibility and trust that ASHAs experience within their communities [33]. This reality may discourage community members from approaching ASHAs as their first contact in the health system. However, understanding CHWs’ perspectives may help to improve counselling and behaviour change outcomes given they are in a better position to contextualize and customize messages for the community.

Another implication of this study is that a top-down approach for identifying and linking vulnerable children to appropriate health services has a high risk of failure because of different perspectives on risk factors between community members and facility staff. Programmatically, ASHAs and ANMs sit at the intersection between community members and facility staff and can link vulnerable and sick children to the care they require. This can be accomplished by strengthening the relationships among community members, CHWs and the health system through: joint ownership and design of CHW programmes, collaborative supervision and constructive feedback, involvement of communities in priority setting, an appropriate and timely package of incentives and an effective monitoring system linking data from communities and the health system.

One of the potential limitations of the study was that during the initial phase of data collection, it was difficult to get clear consensus on vulnerability as there is no specific/local term in Hindi for vulnerability and most children in the study area were at risk of falling ill. To address this issue, the team guided the study participants by probing about the children who have a greater risk of frequently falling sick compared to other children in their community providing insights on a variety of perceived risk factors. Examples of risk were not given to the participants. Rather, the interviewers kept probing responses until no additional responses were obtained. However, the understanding of vulnerability remains highly contextual and the community understanding of specific terms would need to be verified in the development of any further programming that leverages these results.

## Conclusion

The classic risk factors for childhood illness and death identified in household surveys were often perceived as key risk factors by facility staff but not community members. The perspectives of the CHWs bridged these views. CHWs’ role in improving health outcomes at the grassroots level has been recognized globally. CHWs often belong to or live near the communities where they provide their services. In the Indian context, the ASHAs’ broad perceptions of risk factors for childhood illness can be leveraged to identify and focus their activities on the most vulnerable children in the communities they serve, link them to facilities when they become ill and facilitate the development of innovations in program delivery throughout the community-facility continuum.

## Supplementary Information

**Additional file 1.**

**Additional file 2.**

## Data Availability

The data used and/or analyzed during the current study are available from the corresponding author on reasonable request.

## Abbreviations

ANM: Auxiliary Nurse Midwife
ASHA: Accredited Social Health Activist
AWW: Anganwadi Worker
CHC: Community Health Centre
CHW: Community Health Worker
FGD: focus group discussion
HH: head of households
IDI: in-depth interview
MO: medical officer
SN: staff nurse
U5: under-five
UP: Uttar Pradesh
UP-TSU: Uttar Pradesh Technical Support Unit
