# Supplementary material for: Risk factors for childhood illness and death in rural Uttar Pradesh, India: perspectives from the community, community health workers and facility staff
Source: BMC Public Health. 2021 Nov 6;21:2027. doi: 10.1186/s12889-021-12047-2 (PMC8572490; doi:10.1186/s12889-021-12047-2)
Supplement: Supplementary file 1 — Additional file 1. [file 12889_2021_12047_MOESM1_ESM.docx]

**Supplement to: Risk factors for childhood illness and death in rural Uttar Pradesh, India: perspectives from the community, community health workers and facility staff**

**Examples of the Interview Guide (Staff Nurses) and the Focus Group Discussion Guide (Mothers of Under-Five Children)**

Kanchan Srivastava^1^, Ranjana Yadav^1^, Lorine Pelly*^2^, Elisabeth Hamilton^2^, Gaurav Kapoor^1^, Aman Mohan Mishra^1^, Parwez Anis^1^, Maryanne Crockett^2,3^

^1^India Health Action Trust, 404 - 4th Floor, 20-A Ratan Square, Vidhan Sabha Marg, Lucknow, Uttar Pradesh 226001, India

^2^University of Manitoba, Institute for Global Public Health, R070 Med Rehab Building, 771 McDermot Avenue, Winnipeg, Manitoba R3E 0T6, Canada

^3^University of Manitoba, Departments of Pediatrics and Child Health, Medical Microbiology and Infectious Diseases and Community Health Sciences

*Corresponding author

Author Information

Kanchan Srivastava

[kanchan.srivastava29@gmail.com](mailto:kanchan.srivastava29@gmail.com)

Ranjana Yadav

[ranjanaagza@gmail.com](mailto:ranjanaagza@gmail.com)

Lorine Pelly

[lorine.pelly@umanitoba.ca](mailto:lorine.pelly@umanitoba.ca)

Elisabeth Hamilton

[elisabeth.hamilton@umanitoba.ca](mailto:elisabeth.hamilton@umanitoba.ca)

Gaurav Kapoor

[dr.gauravkapoor85@gmail.com](mailto:dr.gauravkapoor85@gmail.com)

Aman Mohan Mishra

[aman.mta@gmail.com](mailto:aman.mta@gmail.com)

Parwez Anis

[parwezanis@gmail.com](mailto:parwezanis@gmail.com)

Maryanne Crockett

Maryanne.Crockett@umanitoba.ca

**Interview Guide**

***Nurses***

**Topic guide:**

- **Preparatory phase**
  - Coordinate with the of IHAT team for organizing interaction with the appropriate respondents
  - Participants: 1 eligible respondent of defined category, 1 research investigator
  - Estimated time: 1-2 hours
  - Materials needed: paper and pen, tape recorder, private space with seating
- **Discussion phase**
  - Greet the participants
  - Introduce yourself and purpose of this interaction
  - Seek consent
  - Once consent received, proceed with interview

**Consent**

*Hello, my name is ____________ and I work with the University of Manitoba and India Health Action Trust. ­­­­Your CHC has been selected for a study on children who are at risk of severe pneumonia and diarrhoea and you have been invited to participate because of your job as an Nurse. During our discussion today, we hope to gain a better understanding of your experience, knowledge, and ideas in identifying and prioritizing children who are at greatest risk of severe illness and death due to pneumonia and diarrhoea. Your participation will help us to improve our program and develop interventions to target the most vulnerable children.*

*We will be recording this discussion in order to remember what we talked about and present your views in a report. No one from your communities will listen to this discussion and your names will not be shared with anyone. We will ensure that the specific information you provide remains anonymous. Is it okay if we record this discussion?* [Review informed consent form so that the participant understands and signs it.]

*Participation in this discussion is completely voluntary and you should not feel obligated to participate. There is no financial compensation for your participation; however, we do hope that you will participate. We want to hear everyone’s voices so please respect each person’s response. There are no right or wrong answers and we hope that you will feel comfortable to speak freely and share your honest opinions.*

*Our interaction today will last between 1-2 hours. Before we begin, does anyone have any questions or concerns? We thank you in advance for giving us your time.*

**Schedule***:*

1. **General background:**

- **Background information of the respondents on – age, experience, role in general and role in maternal and child health**
- **What do you like the most about working with families and children?**

**B. Perceived prevalence of children U5 health problems in your CHC / community:**

- **What kind of health problems generally you see in children under – 5 in your CHC? (Probe for pneumonia and diarrhoea)**
- **In your whole tenure of service so far, have your seen any case of apidemic or high incidence of P & D in your children U 5? Probe for prevalence**
- **Do you think that pneumonia and diarrhoea are problem in your CHC? Probe for prevalence**
- **Have you ever come across any case of pneumonia in your CHC?**
- **Have any children in your area suffered from pneumonia in last few months? (Probe for her children)**
- **Have you ever come across any case of diarrhoea in your CHC?**
- **Have any children in your area suffered from diarrhoea in last few months?**

**C. Perceived vulnerability: (Probe for her operational area)**

- **What do you think are the reasons that children become ill with pneumonia and diarrhoea? (Probe for her area)**
- **Which is the most vulnerable time for pneumonia? Why?**
- **Which is the most vulnerable season for pneumonia? Why?**
- **Which types of babies have more chances for having pneumonia? Why?**
- **Which is the most vulnerable time for diarrhoea? Why?**
- **Which is the most vulnerable season for diarrhoea? Why?**
- **Which types of babies have more chances for having diarrhoea? Why?**
- **Probe for role of gender in vulnerability?**
- **Probe for role of ‘*kuposhan’* in pneumonia and diarrhoea? Why? How much?**

**D. Knowledge and understanding of the household member around pneumonia and diarrhoea?**

- **What are the differences between a child at risk for severe pneumonia or diarrhoea and any other child not at risk for severe pneumonia and diarrhoea in your area?**
- **How important is it to identify the children in your area who are at risk for severe pneumonia and diarrhoea before they become ill?**
- ***What training did you receive, when did you receive, duration etc? (For nurses)***

**E. Experience with respect to prevention and management of pneumonia and diarrhoea**

- **What is the first thing you do when you think that the child is ill?**
- **Probe for: what is done at the CHC level, ASHA level and ANM level to prevent pneumonia?**
- **Probe for: what is done at the CHC level, ASHA level and ANM level to manage pneumonia?**
- **Recall any case of pneumonia; and how you managed or helped to manage the illness? What did you do? (In case of no case, ask them what should be done?**
- **Probe for: what is done at the CHC level, ASHA level and ANM level to prevent diarrhoea?**
- **Probe for: what is done at the CHC level, ASHA level and ANM level to manage diarrhoea?**
- **Recall any case of diarrhoea; and how you managed or helped to manage the illness? What did you do? (In case of no case, ask them what should be done?**
- **Probe for usage of ORS, rota virus and pneumococcal vaccines? In which children? When? Administration of vaccines related issues? During the year when they require high demand of each of these? Why?**
- **Based on your experience, have there been any reductions in the incidence of P & D in Uttar Pradesh? If yes, how it achieved? If no, why?**

**F. Challenges in identification and management of P & D?**

- **What challenges are being faced at your level in timely identification of baby who has the risk of having pneumonia?**
- **What challenges are being faced at your level in timely identification of baby who has the risk of having diarrhoea?**
- **What can make it difficult to access health services? (Probe about transportation, costs, family decision-making, and availability of health providers.)**

**G. Solutions/ suggestions in improving timely identification and prioritizing care of P & D vulnerable houses?**

- **What change is needed at the level of following in timely identification of pneumonia and diarrhoea cases/ high risk households where likelihood for p& D is high? How we will pin point those children as early as possible?**
  - **MOTHER**
  - **FAMILY**
  - **VHND**
  - **AWW**
  - **ANM**
  - **CHC**
  - **ASHA**
- **In what ways we can sensitize the high risk households to prioritize care of baby who has the risk of having diarrhoea?**
- **Do you have any idea about activities that would help identify the children in your village who are at risk for severe pneumonia and diarrhoea? How could these ideas be incorporated into your current work?**
- **What could make these activities successful in your village? Can you imagine what barriers exist that would impact these activities? (probe if this would cause a burden on their time and responsibilities.)**
- **We have talk about this topic with other CHWs, ANMs and CHC staff. They have suggested a few ideas for interventions (list ideas one at a time). What interventions / idea would be best for you and why? What would you need to implement these activities and overcome those barriers?**

**Closing**

*Would anyone like to add anything that is important and we haven’t spoken about today?*

*We want to thank you for your patience and your time in participating in the discussion. The discussion was extremely valuable and we have learned a lot from you. Your responses will help inform future interventions. We hope that you also enjoyed participating. Thank you and goodbye!*

**Focus Group Discussion Guide**

***Mothers of Children Under 5***

**Topic guide:**

- **Preparatory phase**
  - Coordinate with the MOIC/ BCPM/ ASHA Sangini with the help of IHAT staff for organizing FGD with the appropriate respondents
  - Participants: 6 to 8 respondents of defined category e.g. mothers of children under 5, 1 facilitator, 1 note taker
  - Estimated time: 1-2 hours
  - Materials needed: paper and pen, tape recorder, private space with seating
- **Discussion phase**
  - Greet the participants
  - Introduce yourself and purpose of this interaction
  - Seek consent
  - Once consent received, proceed with discussion

**Consent**

*Hello, my name is ____________ and I work with the University of Manitoba and India Health Action Trust. ­­­­Your village has been selected for a study on children who are at risk of severe pneumonia and diarrhoea and you have been invited to participate because you are all mothers with children under 5 years of age. During our discussion today, we hope to gain a better understanding of your knowledge about pneumonia and diarrhoea and your experiences with having young children and using the health system when they are ill. Your participation will help us to improve our program and develop interventions to target the most vulnerable children.*

*We will be recording this discussion in order to remember what we talked about and present your views in a report. My colleague is also going to take some notes about what you share with us. No one from your communities will listen to this discussion and your names will not be shared with anyone. We will ensure that the specific information you provide remains anonymous. Is it okay if we record this discussion?*[Review informed consent form so that each participant understands and signs it.]

*Participation in this discussion is completely voluntary and you should not feel obligated to participate. There is no financial compensation for your participation; however, we do hope that you will participate. We want to hear everyone’s voices so please respect each person’s response. There are no right or wrong answers and we hope that you will feel comfortable to speak freely and share your honest opinions.*

*Our discussion today will last between 1-2 hours. Before we begin, does anyone have any questions or concerns? We thank you in advance for giving us your time.*

**Introduction**

*Beginning with the facilitator and note taker, ask participants to introduce themselves. Introductions should include name, age, occupation, and number of children (including number of children under 5) in the household. The note taker should capture all introductory information (except names) from each participant.*

**Schedule***:*

1. **General background:**

- **Background information of the respondents on – age, experience, role in general and role in maternal and child health**

**B. Perceived prevalence of children U5 health problems in your family / community:**

- **What kind of health problems generally you see in children under – 5 in your village and in your family? (Probe for pneumonia and diarrhoea)**
- **Do you think that pneumonia and diarrhoea are problem in your village? Probe for prevalence**
- **Have you ever come across any case of pneumonia in your village?**
- **Have any children in your family suffered from pneumonia in last few months? (Probe for her children)**
- **Have you ever come across any case of diarrhoea in your village?**
- **Have any children in your family suffered from diarrhoea in last few months or year? (Probe for her children)**

**C. Perceived vulnerability:**

- **What do you think are the reasons that children in your family/ village become ill with pneumonia and diarrhoea?**
- **Which is the most vulnerable time for pneumonia? Why?**
- **Which is the most vulnerable season for pneumonia in your village? Why?**
- **Which types of babies have more chances for having pneumonia? Why?**
- **Which is the most vulnerable time for diarrhoea? Why?**
- **Which is the most vulnerable season for diarrhoea in your village? Why?**
- **Which types of babies have more chances for having diarrhoea? Why?**
- **Probe for role of gender in vulnerability?**
- **Probe for role of ‘*kuposhan’* in pneumonia and diarrhoea? Why? How much?**

**D. Knowledge and understanding of the household member around pneumonia and diarrhoea?**

- **How do you recognize that the baby / child have got pneumonia? What is pneumonia according to you? Understanding on symptoms, grades based on severity? How do you refer to pneumonia in your family and area? Local names, identification? How do you assess whether its pneumonia or some other problems?**
- **How do you recognize that the baby / child have got diarrhoea? What is diarrhoea according to you? Understanding on symptoms, grades based on severity? How do you refer to diarrhoea in your family and area? Local names, identification? How do you assess whether its diarrhoea or some other problems?**
- **What do you notice about your child before he /she becomes ill with pneumonia and diarrhoea?**

**E. Experience with respect to prevention and management of pneumonia and diarrhoea**

- **What is the first thing you do when you think your child is ill?**
- **Where do you take her/him for care?(probe if they go to different providers for different illness and also if visiting an ASHA/ANM is an option and why/why not.)**
- **Probe for: what is done at the community level, ASHA level and ANM level to prevent pneumonia?**
- **Recall any case of pneumonia; and how you managed or helped to manage the illness? What did you do? (In case of no case, ask them what should be done?**
- **Probe for: what is done at the community level, ASHA level and ANM level to prevent diarrhoea?**
- **Recall any case of diarrhoea; and how you managed or helped to manage the illness? What did you do? (In case of no case, ask them what should be done?**

**F. Challenges in identification and management of P & D?**

- **What challenges are being faced in timely identification of baby who has the risk of having pneumonia?**
- **What challenges are being faced in timely identification of baby who has the risk of having diarrhoea?**
- **What can make it difficult to access health services? (Probe about transportation, costs, family decision-making, and availability of health providers.)**

**G. Solutions/ suggestions in improving timely identification and prioritizing care of P & D vulnerable houses?**

- **What change is needed at the level of following in timely identification of pneumonia and diarrhoea cases/ high risk households where likelihood for p& D is high? How we will pin point those children as early as possible?**
  - **MOTHER**
  - **FAMILY**
  - **VHND**
  - **AWW**
  - **ANM**
  - **CHC**
  - **ASHA**
- **In what ways we can sensitize the high risk households to prioritize care of baby who has the risk of having diarrhoea?**
- **Do you have any idea about activities that would help identify the children in your village who are at risk for severe pneumonia and diarrhoea?**
- **What could make these activities successful in your village?**
- **We have talk about this topic with ASHA, ANMs and CHC staff. They have suggested a few ideas for interventions (list ideas one at a time). What interventions/idea would be best for you and why?**

**Closing**

*Would anyone like to add anything that is important and we haven’t spoken about today?*

*We want to thank you for your patience and your time in participating in the discussion. The discussion was extremely valuable and we have learned a lot from you. Your responses will help inform future interventions. We hope that you also enjoyed participating. Thank you and goodbye!*
