# Supplementary material for: Risk factors for childhood illness and death in rural Uttar Pradesh, India: perspectives from the community, community health workers and facility staff
Source: BMC Public Health. 2021 Nov 6;21:2027. doi: 10.1186/s12889-021-12047-2 (PMC8572490; doi:10.1186/s12889-021-12047-2)
Supplement: Supplementary file 2 — Additional file 2. [file 12889_2021_12047_MOESM2_ESM.docx]

**Supplement to: Risk factors for childhood illness and death in rural Uttar Pradesh, India: perspectives from the community, community health workers and facility staff**

**Codes and subcodes**

Kanchan Srivastava^1^, Ranjana Yadav^1^, Lorine Pelly*^2^, Elisabeth Hamilton^2^, Gaurav Kapoor^1^, Aman Mohan Mishra^1^, Parwez Anis^1^, Maryanne Crockett^2,3^

^1^India Health Action Trust, 404 - 4th Floor, 20-A Ratan Square, Vidhan Sabha Marg, Lucknow, Uttar Pradesh 226001, India

^2^University of Manitoba, Institute for Global Public Health, R070 Med Rehab Building, 771 McDermot Avenue, Winnipeg, Manitoba R3E 0T6, Canada

^3^University of Manitoba, Departments of Pediatrics and Child Health, Medical Microbiology and Infectious Diseases and Community Health Sciences

*Corresponding author

Author Information

Kanchan Srivastava

[kanchan.srivastava29@gmail.com](mailto:kanchan.srivastava29@gmail.com)

Ranjana Yadav

[ranjanaagza@gmail.com](mailto:ranjanaagza@gmail.com)

Lorine Pelly

[lorine.pelly@umanitoba.ca](mailto:lorine.pelly@umanitoba.ca)

Elisabeth Hamilton

[elisabeth.hamilton@umanitoba.ca](mailto:elisabeth.hamilton@umanitoba.ca)

Gaurav Kapoor

[dr.gauravkapoor85@gmail.com](mailto:dr.gauravkapoor85@gmail.com)

Aman Mohan Mishra

[aman.mta@gmail.com](mailto:aman.mta@gmail.com)

Parwez Anis

[parwezanis@gmail.com](mailto:parwezanis@gmail.com)

Maryanne Crockett

Maryanne.Crockett@umanitoba.ca

Supplementary Table 1. Codes and subcodes

| Codes | Subcodes |
| --- | --- |
| Action taken to prevent and manage P&D | - Management and Prevention Diarrhoea - Management and Prevention Pneumonia - Prevention and Management-Diarrhea and Pneumonia - Management and Prevention-In General - Management and Prevention Cold and Cough - Management –Decision making - Management –Counselling - Management-Precaution - ORS Preparation |
| Identification of health problem | - Diarrhoea - Pneumonia - Cold - Malnourishment - In General - Identification-Strategy |
| Perceived prevalence | - General perceived illness - Reason of prevalence - Perceived prevalence - Mostly prevalent - Other perceived |
| Understanding of health problems among children under 5 | - Understanding of Disease: an overview - Understanding of Diarrhoea - Understanding of Pneumonia - Perceived Vulnerable among Under5 - Local Name |
| Perceived risk factor | - Occupation/work related factors - Caste/religion - Household risk factors - Poor families - Literacy - Location of habitation - Notion of hot/cold - Seasonality - Lack of adequate nutrition - Environmental - Lack of attentiveness by caregiver - Health and wellbeing of mother - Hygiene - Gender difference |
